# Polymorphisms Involved in Response to Biological Agents Used in Rheumatoid Arthritis

**DOI:** 10.3390/biom10091203

**Published:** 2020-08-19

**Authors:** Giovanni Pallio, Federica Mannino, Natasha Irrera, Ali H. Eid, Francesco Squadrito, Alessandra Bitto

**Affiliations:** 1 Department of Clinical and Experimental Medicine, University of Messina, Via C. Valeria, 98125 Messina, Italy; gpallio@unime.it (G.P.); fmannino@unime.it (F.M.); nirrera@unime.it (N.I.); fsquadrito@unime.it (F.S.); 2 Department of Pharmacology and Toxicology, American University of Beirut, Bliss Street, Riad El Solh, 1107-2020 Beirut, Lebanon; ae81@aub.edu.lb

**Keywords:** polymorphism, rheumatoid arthritis, TNF-α, IL-6, IL-1, CD20, CD80, CD86

## Abstract

Rheumatoid arthritis (RA) is a systemic disease that leads to joint destruction. During the last decade, the therapy of RA has been principally based on biological drugs. Although the efficacy of biological therapy has been established, patients demonstrated a high heterogeneity in clinical response to treatment. Several genetic polymorphisms play a part in the different response to biological drugs. This review summarizes the pharmacogenetics of biological agents approved for clinical RA treatment. We reviewed PubMed papers published over the past 20 years (2000–2020), inserting as the search term “rheumatoid arthritis and polymorphisms”. Despite some studies showing important correlations between genetic polymorphisms and response to biological therapy in RA patients, most of these findings are still lacking and inconsistent. The personalized treatment according to a pharmacogenetics approach is promising but the available pharmacogenetics data on biological treatment in RA are not adequate and reliable to recommend pharmacogenetic tests before starting biological therapy in RA patients.

## 1. Introduction 

Rheumatoid arthritis (RA) is a crippling chronic inflammatory and autoimmune disease. It is characterized by the presence of autoantibodies, systemic inflammation, as well as damage at synovial joints that consequently leads to damage in the articular cartilage. RA affects 0.41–0.52% of adults in developed countries with 40 per 100,000 new cases annually. Interestingly, this disease is more prevalent in women and elderly people, with smoking being the primary environmental risk factor in the onset of this disease [1,2]. The pathogenesis of RA is characterized by infiltration of fibroblast-like and macrophage-like synoviocytes, macrophages, several populations of T and B cells into the synovial tissue, which release pro-inflammatory products (e.g., tumor necrosis factor TNF-α) but also increase the synovial fluid volume and activate a series of immune responses that lead to the production of autoantibodies that further implement the injury [3,4]. The structurally altered synovia loses its protective role and bone erosion appears, further corrupting the joint. The overproduction of many pro-inflammatory cytokines such as interleukin 1β and TNF-α, which evoke persistent inflammation and joint destruction, and in particular of IL-6, may contribute not only to the progression of local disease but also to the concomitant hypergammaglobulinemia and thrombocytosis [5]. Several classes of drugs are currently employed for the treatment or management of RA. These include analgesics and non-steroidal anti-inflammatory drugs (NSAIDs) that are primarily used to ameliorate the presented symptoms. Nonetheless, these drugs have lost their historical role as first-line treatment largely due to their limited efficacies, inability to modify the long-term course of disease continuum, as well as gastrointestinal and cardiac adverse effects [6]. The other class is disease-modifying antirheumatic drugs (DMARDs), which represent a heterogeneous collection of agents grouped together for use in this disease. These drugs decrease joint-swelling and pain, reduce acute-phase markers and limit progressive joint damage [7].

Before the advent of biologic therapy, most RA cases were treated using a combination of DMARDs; however, for refractory and severe cases, anti-TNF therapy has become a foundation of RA treatment. These anti-TNF biological drugs are divided into two classes: the TNF-receptor fusion protein (certolizumab, etanercept), which prevents TNF action by binding to its cell surface receptor, and TNF-binding monoclonal antibodies (adalimumab, golimumab, and infliximab). Both classes share similar underlying molecular mechanisms, which could then explain their similar effects in modulating inflammatory cytokines levels, complement activation, lymphocyte recruitment, and apoptosis [8,9].

TNF-inhibitors show high efficacy in DMARDs non-responder patients [10]. However, around 30% of patients do not display clinical improvement following administration of a TNF-inhibitor. RA is a polygenic and multifactorial disease, and thus it is only expected that response to treatment may be influenced by genetic factors. In this context, identifying polymorphisms that can lead to inefficacy of treatment or side effects may be warranted. During recent years, several studies showed contradictory results about the potential association of anti-TNF blocker’s response and polymorphisms both in the TNF gene (positions −308, −238, −857, and 489) [11,12,13] or other related genes such as TNF receptors (TNFR1 and TNFR2) [14]. Furthermore, as previously described, IL-6 inflammatory cascade is involved in RA pathogenesis and its expression has been shown to be modulated by −174 G/C polymorphisms in the promoter regions of the IL-6 gene. In particular, carriers of the C allele showed higher serum levels of IL-6 than carriers of G/G genotype in both the general population and RA patients [15]. Contextually, compared with the G/G genotype, C/C or G/C genotypes are associated with higher severity of RA, due to increased IL-6 production which in turn reduces responsiveness to biological agent therapy [16,17]. Moreover, patients with single nucleotide polymorphism (SNP) in +1970 position of the gene coding for IL-1 receptor (IL-1R) exhibited higher levels of this receptor and were more susceptible to developing a severe form of AR [18]. This may explain how the C/C genotype in this SNP could influence the efficacy of biological agent. 

A role for other co-stimulatory molecules, CTLA-4, CD28, CD80, and CD86 in the development of rheumatic diseases, has been established. However, polymorphisms of these genes were not significantly associated with RA development or response to treatment [19,20]. Additionally, CD20 antigen expressed in B cells is involved in the B-cell depletion with several mechanisms including complement-dependent cytotoxicity and antibody-dependent cell-mediated cytotoxicity, which are influenced by the different affinities of Fc receptor to the Fc fragment of IgG. It has been reported that FCGR3A -158V > F polymorphism was related to the affinity of Fc receptor for IgG. -158F carrier bound significantly less IgG, than −158V carrier. Consequently, this variation in the affinity of Fc receptor for IgG may affect the differences in clinical response of RA patients to rituximab, a monoclonal antibody against CD20 [21].

In light of all these observations, we review the pharmacogenetic biological treatment of RA, focusing on TNF-α, IL6, and anti-CD20.

## 2. Methodology

The database used to retrieve the papers was PubMed and as search terms used were “tumor necrosis factor-alpha and polymorphism and rheumatoid arthritis”; “IL-6 and polymorphism and rheumatoid arthritis”; “IL-1 and polymorphism and rheumatoid arthritis”; “CD80 or CD86 or abatacept and polymorphism and rheumatoid arthritis”; “CD20 or rituximab and polymorphism and rheumatoid arthritis”; “Tofacitinib or upadacitinib or baricitinib and polymorphism and rheumatoid arthritis”. Papers published in the last 20 years (2000–2020) showing a significant difference in the response to drug treatments were included to provide a broader overview of those genetic variants that affect biological treatment in RA. Papers focusing on SNPs without a significant correlation with clinical outcomes (Disease Activity Score 28 and/or EULAR response) were excluded. Most of the cited papers have been published by European researchers (90%), although papers from Asian study groups (10%) have been also taken into account.

## 3. Pharmacogenetics and Biological Agents Used in Rheumatoid Arthritis

### 3.1. Pharmacogenetics of TNF-α and TNFR

In the context of RA treatment, several studies (Table 1) have examined the role of SNPs in the TNF-α gene as a marker of poor response. For instance, Mugnier et al. genotyped 59 patients with RA, stratified them according to their genotype and compared the effect of infliximab treatment after 22 weeks using Disease Activity Score 28 (DAS28 score). The average improvement in the DAS28 score was 1.24 in patients with the A/A and A/G genotype and 2.29 in the G/G patients (*p* = 0.029). These data suggest that patients with a TNF-α −308 G/G genotype are better infliximab responders than patients with A/A or A/G genotypes [22]. In a prospective study of 22 patients with RA who were cured with infliximab, and after 24.8 months of infliximab treatment, patients with the −308 G/G genotype showed a decrease in the DAS28 score of 2.4 ± 0.6, whereas the −308 A/G group had an increase of 0.12 ± 0.18 (*p* < 0.01). These results confirm that RA patients with -308 G/G genotype had a significantly better response to infliximab than patients with −308 A/G genotype [23]. Another study genotyped 81 patients with RA treated with adalimumab and evaluated the clinical improvement after 24 weeks of treatment. The DAS28 score improvement at week 24 was 2.5 ± 1.3 in the G/G group and 1.8 ± 1.3 in the G/A group (*p* = 0.04) [24]. Similarly, others have genotyped 86 patients with RA treated with etanercept and compared clinical response between group with A/G genotype and G/G genotype after 6 months of treatment using DAS28 score. DAS28 improvement was 1.69 ± 1.31 in the group of patients with A/G genotype and 2.23 ± 1.19 in the group with G/G genotype (*p* = 0.098) [25].

Studies employing infliximab, adalimumab, or etanercept have also been conducted in genotyped patients suffering from RA. After 24 weeks of treatment, the average improvement in the DAS28 score was 0.83 in the A/A, 1.50 in the A/G and 2.64 in the G/G group respectively (*p* < 0.0001). These results confirmed that patients with TNF-α-308 G/G genotype are better responders to anti-TNF-alpha treatment than those with A/A or A/G genotypes independently to biological agent used [26]. It was interesting then to determine if TNF-α SNPs affect response to treatment. Indeed, it was then showed that a relationship between SNP at TNF-238 or TNF-308 and the different response to treatment founded on the agent used. In particular, patients carrying −308A/A genotype poorly respond to etanercept (*p* = 0.001), whereas this SNP does not associate with response to infliximab [8]. Conversely, the G/A genotype at TNF-238 was associated with a poorer response to infliximab (*p* = 0.028), but not etanercept [8].

There have been several meta-analyses that determined the association between various SNPs and responsiveness to TNF-α inhibitors. One of these studies showed a significant association between the TNF-α promoter −308 A/G polymorphism and responsiveness to anti-TNF therapy, indicating that RA patients with the A allele have a lesser response to anti-TNF agent than individuals with the G allele [27]. Another meta-analysis of nine studies with a total of 692 patients showed that the presence of an A allele in position -308 significantly decreases the response to TNF-α inhibitors [28]. Likewise, a meta-analysis of 15 studies with a total of 2127 patients concluded that carrying the G allele in position −308 is associated with a stronger response to biological treatment than the A/A genotype [29].

Various other studies have looked into the effects of other SNPs. For instance, a study looking at the −857 C/T polymorphism in 70 patients treated with etanercept showed that RA patients whit the T allele (T/T or C/T) respond better to etanercept therapy than homozygotes for the C allele (C/C). This indicates that this SNP may become a possible genetic marker for predicting anti-TNF therapeutic effects [30]. Studying three TNF-α gene polymorphisms (−238 A/G, -308 A/G and -857 C/T) showed that the −238G, −308G, or −857C haplotype in a homozygous form was significantly associated with a lower ACR50 response to adalimumab [31].

SNPs in the TNFR are also associated with responsiveness. Indeed, in RA patients on anti-TNF-α therapy after 3 months, the TNFR1 36 A/A genotype was associated with a worse DAS-based European League Against Rheumatism (EULAR) response than A/G or G/G genotype to (*p* = 0.04) [14]. Moreover, RA patients with TNFR2 rs1061622 T allele had a worst response to anti-TNF therapy compared to patients with the TNFR2 rs1061622 G allele (*p* = 0.045) [32]. Similarly, TNFR2 3397C/C, TNFR2 rs1061622 G/G, and TNFR2 rs1061631 A/A genotypes had an increased risk of having a poorer response to anti-TNF drugs (*p* = 0.014, *p* = 0.0085 and *p* = 0.028 respectively) [33]. Patients with TNFR2 676 T/G genotype was significantly associated with lower ACR response compared to patients with 676 T/T genotype, after 3 and 12 months of treatment [34].

There are some studies that looked at a combination of SNPs in both the receptor and the TNF gene. In a study that enrolled 58 RA patients receiving infliximab, six polymorphisms, 36A > G in TNFR1; 676T > G in TNFR2; −857C > T, −308G > A, −238G > A and 489G > A in TNF-α gene and their relationship to therapeutic efficacy were determined. This study showed that a combination of 676T > G (TNFR2) and −857C > T (TNF-α) could influence infliximab therapeutic efficacy in RA patients [35]. Similarly, in 280 RA patients treated with TNF-inhibitors, an association study of five SNPs in TNF-α and TNF-receptor encoding genes (TNF-α: −308G > A, −238G > A, −857C > T; TNFR1 36A > G; TNFR2 676T > G) was investigated. After 3 months of treatment, patients with the TNFR1 36 A/A genotype achieved a good EULAR response compared to patients carrying the G allele (*p* = 0.011). After 6 months low disease activity was observed in patients with TNFR1 36 A/A genotype than patients with G/G genotype (*p* = 0.04). Moreover, after 6 months DAS28 score was significantly lower in the subgroup of patients with TNF-α-857 T/T genotype compared to C allele carriers (*p* = 0.012) [36].

In summary, treatments are affected mainly by the presence of the G allele in −308 TNF-α polymorphism carriers disregarding of the anti-TNF used (Table 1). Moreover, the presence of polymorphic sites on TNF-α receptors was also associated with a better response of treated patients, suggesting that a pre-evaluation could be useful for prescribing the most useful drug, according to patient’s genetic profile.

### 3.2. Pharmacogenetics of Interleukin-6 and Interleukin-6R and Their Influence on Anti-TNF and Anti-CD20 Therapy in RA

The IL-6 inflammatory cascade is involved in RA pathogenesis. Hence, antibodies targeting IL-6R, such as tocilizumab and sarilumab, were used for the treatment of RA. Although limited, there are a few studies that investigated the role of genetic variations in response to these biological treatments. For instance, three IL-6R, SNPs 8435A > G rs12083537, 54302A > C rs8192284 and 59752T > C rs4329505, were investigated in 79 RA patients. Results showed that the presence of the AAC haplotype (for rs12083537, rs8192284, and rs4329505) was associated with a poor SJC (swollen joint count) and EULAR response (*p* = 0.00004; *p* = 0.05 respectively) [37]. Another study investigated the influence of rs12083537, rs2228145, rs4329505 and rs11265618 SNPs in IL-6R on response to tocilizumab in 77 RA patients [38]. This study showed that the A/A genotype for rs12083537 (*p* = 0.004) and C/C genotype for rs11265618 (*p* = 0.004) had better LDA (low disease activity) response after 12 months of treatment [38]. Moreover, a recent paper confirmed that the response to tocilizumab was associated with SNPs in the IL-6R gene. Patients with A/A genotype for rs12083537 showing a significantly better response than homozygous or heterozygous patients with the G allele (*p* = 0.018) [39].

Polymorphisms in IL-6 promoter may also affect response to anti-TNF agent. For instance, in a cohort of 77 RA patients, a relationship between disease activity and the -174G > C IL-6 gene promoter polymorphism before and after etanercept therapy was noted. Indeed, after 12 months of treatment, the percentage of patients responding to treatment was significantly higher in patients with G/G genotype (95.7%) compared to patients with the G/C (75.6%) or C/C (44.4%) genotype (*p* = 0.006) [17]. These results were confirmed by another study showing that patients receiving anti-TNF therapy had a significant correlation between the IL-6 –174G allele and a good or moderate EULAR response at 12, 18 and 24 months [40]. Furthermore, this was supported by a meta-analysis which showed that patients carrying the IL-6 −174C allele have a poorer response to anti-TNF therapy for RA [41]. Contextually, after 12 months of etanercept therapy, patients with the combined IL-6 −174 G/G and TNF-α −308 G/G genotype were, more frequently, responders to treatment (DAS28 improvement > 1.2) compared to patients with other combined genotypes (*p* = 0.022) [42].

Rituximab, a chimeric monoclonal antibody against CD20, has also been used to treat RA patients. A relationship between various SNPs and responsiveness to rituximab in RA patients has been reported. In particular, polymorphisms in the promoter of IL-6 may also affect response to rituximab. Moreover, C/C homozygosis of the −174 IL-6 promoter polymorphism appears to be a predictor of no response to rituximab in RA patients [43,44].

In summary, IL-6R 8435 A/A genotype was associated with better response to tocilizumab. Moreover, IL-6 −174 G/G genotype was associated with better response to anti-TNF agent (infliximab, adalimumab, etanercept) and anti-CD20 rituximab (Table 2), suggesting that a pharmacogenetic pre-evaluation of patients could be useful for a targeted treatment of RA patients.

### 3.3. Pharmacogenetics and Anti-CD20 Treatment

Several studies demonstrated that the efficacy of anti-CD20 treatment varies inter-individually. For instance, FCGR3A −158V allele was significantly associated with a higher response rate to rituximab treatment in patients with RA who did not respond or tolerate anti-TNF therapy [45]. Similarly, in another study, heterozygous patients (FCGR3A-158VF) showed higher rate of responsiveness to rituximab [46]. Moreover, in −158 V/V patients the response rate differed between male and female patients (*p* = 0.036) demonstrating that the impact of −158 V/V genotype on rituximab response may be influenced by sex [46]. In another study involving 212 RA patients, the FCGR3A −158 V/V genotype was associated with higher ratio (89.5%) of EULAR response (good or moderate) at 6 months compared to V/F (66%) or F/F (66.2%) [47]. A low disease activity score was achieved in patients with V/V (62.5% *p* = 0.030) or V/F (64.7% *p* = 0.015) compared with F/F subjects (30.0%) [48].

Other SNPs of various genes have also been studied in the context of RA and rituximab. One study analyzed 13 SNPs located in genes coding for IL-10, LTA, TGFβ1, TNF-α, TNFR2, −C5 TRAF1, STAT4, TNFAIP3, and PTPN22. Only TGFβ1 10 C/T genotype and TGFβ1 25 G/C genotype were associated with a better clinical response [49]. Moreover, polymorphisms on B-cell-activating factor (BAFF) gene, coding for a cytokine with an important role in B-cell stimulation were associated with rituximab response. Indeed, in a study involving 115 RA patients, BAFF −871 C>T polymorphism correlation with EULAR response after 24 weeks of rituximab treatment was evaluated. This study showed that the C/C genotype was significantly associated with a higher response rate (92%) than the T/T genotype (64%). The positive association between the presence of a C allele and good response to rituximab treatment was further confirmed in a multivariate analysis, where carriage of this allele was independently correlated with higher response to rituximab [50]. Additionally, the effects of TTTT BAFF haplotype on response to rituximab therapy in RA patients showed that this haplotype was more recurrent in good (41.9%) than in moderate responders (24.1%) [51]. Furthermore, multivariate analysis confirmed that TTTT BAFF promoter haplotype is an independent marker of good response to rituximab treatment in RA patients [51]. Moreover, Jiménez Morales and colleagues investigated the influence of FCGR2A/FCGR3A gene polymorphisms in 55 RA patients treated with rituximab according to low disease activity rate and DAS 28 score at 6, 12, 18 months. Carriers of the FCGR2A rs1801274-TT genotype had higher EULAR response at 6 (*p* = 0.035) and 12 months (*p* = 0.066) and a greater improvement in DAS28 score at 12 (*p* = 0.098) and 18 months (*p* = 0.025). The FCGR3A rs396991-G allele was associated with improved low disease activity rate (*p* = 0.077) and a greater improvement in DAS28 score (*p* = 0.021) at 18 months [52].

In summary, rituximab efficacy is affected mainly by the presence of FCGR3A −158V allele. Moreover, BAFF −871 C/C genotype was also associated with a better response of RA patients treated with rituximab, suggesting that a pharmacogenetics pre-evaluation could be useful to improve the efficacy of these biological treatments in RA patients (Table 2).

### 3.4. Pharmacogenetics of CD80 and CD86 

Abatacept is a recombinant fusion protein composed of the Fc region of the immunoglobulin IgG1 fused to the extracellular domain of cytotoxic T-lymphocyte-associated antigen 4 (CTLA4). CTLA4-Ig acts as a competitive inhibitor of CD28 on the T-cell surface by binding with either CD80 (ligand B7-1) or CD86 (ligand B7-2) on the antigen-presenting cell [53]. Blocking CD80 or CD86 prevents the second signal, without which T-cell activation would be hindered. Abatacept is clinically used for the treatment of RA patients with unsatisfactory response to traditional DMARDs or TNF antagonists. To date, only one study examined the role of genetic polymorphisms in the treatment efficacy in RA patients; however, the results are of no clinical relevance (Table 2). In fact, SNPs in CTLA4 gene do not predict the response to abatacept [20].

### 3.5. Pharmacogenetics of Interleukin-1 

Anakinra is a recombinant IL-1 receptor (IL1-R) antagonist. To the best of our knowledge, only one study examined the association between IL-1 genotype and the treatment response to anti-IL-1 therapy. Importantly, a significant association was found between carriage of the minor allele IL-1α (+4845) and response to treatment [54]. Moreover, a slighter association was found for carriage of the minor allele IL-1β (+3954) and anakinra treatment. Interestingly, a highly significant association between treatment outcome and the haplotype IL1 α (+4845) and IL1 β (+3954) was noted [54].

### 3.6. Pharmacogenetics of Janus Kinase (JAK)

Tofacitinib, upadacitinib and baricitinib are orally administered Janus kinase (JAK) inhibitor, used to treat RA. To date there are no studies examining the associations between SNPs and treatment outcome of RA patients with these biological drugs.

## 4. Conclusions

Several pharmacogenetic studies investigated associations between SNPs and response to biological treatment in RA. The candidate genes for SNPs study were chosen from the genes involved in RA cytokines signaling cascade. Numerous papers have reported that some SNPs might have a linkage to biological treatment response and could have a potential role as possible predictors of response in particular for TNF-α inhibitors, anti-IL-6R drugs, and CD20 inhibitor. On the other hand, these associations have been rarely confirmed in large cohorts of patients or meta-analysis studies to indicate the weakness of these associations. Moreover, to date there are few studies investigating the associations between SNPs and clinical outcome of RA patients treated with anti-CD80 and CD86, anti-Interleukin-1R, or Janus kinase (JAK) inhibitors. Presently, from the clinical perspective, the most suitable way to move forward in the management of RA patients is to improve TNF-α pharmacogenomics, considering in the next studies bias such as ethnicity, different criteria to define clinical responder or different kinds of biological agents used that could influence the clinical results and the scientific value of investigations. From these speculations arises the importance of using genetic analysis to switch the personalized biological therapy of RA patients from an empiric strategy into a sound and cost-effective clinical practice.

## Figures and Tables

**Table 1:** Summary of studies on pharmacogenetics of anti-TNF treatment in RA.

Study	Polymorphic Locus	Biological Agent	Clinical Effects
Mugnier et al. 2003	TNF-α −308	Infliximab	TNF-α −308 G/G was associated with better response than TNF-α -308 G/A or A/A
Fonseca et al. 2005	TNF-α −308	Infliximab	TNF-α −308 G/G was associated with better response than TNF-α −308 G/A
Cuchacovich et al. 2006	TNF-α −308	Adalimumab	TNF-α −308 G/G was associated with better response than TNF-α −308 G/A
Guis et al. 2007	TNF-α −308	Etanercept	TNF-α −308 G/G was associated with better response than TNF-α −308 G/A
Seitz et al. 2007	TNF-α −308	Infliximab–Adalimumab–Etanercept	TNF-α −308 G/G was associated with better response than A/A or A/G independently to biological agent used
Kang et al. 2005	TNF-α −857	Etanercept	TNF-α −857 T/T or C/T were associated with better response than TNF-α −857 C/C
Miceli-Richard et al. 2008	TNF-α −238 TNF-α −308 TNF-α −857	Adalimumab	TNF-α −238G/−308G/−857C haplotype in a homozygous form was associated with a lower response
Ongaro et al. 2008	TNFR2	Infliximab–Adalimumab–Etanercept	TNFR2 676 T/T was associated with a better response compared to 676 T/G
Swierkot et al. 2015	TNF-α FR1 TNFR2	Infliximab–Adalimumab–Etanercept	TNFR1 36 A/A was associated with better response than G/G. TNF-α −857 T/T showed better response than C allele carriers

**Table 2:** Summary of studies on pharmacogenetics of anti-Interleukin-6, anti-CD20, anti-Interleukin-1 treatments in RA.

Study	Polymorphic Locus	Biological Agent	Clinical Effects
Enevold et al. 2014	IL-6R rs12083537; rs8192284; rs4329505	Tocilizumab	AAC-haplotype for rs12083537; rs2228145; rs4329505, was associated with a poor SJC and EULAR response
Maldonado-Montoro et al. 2016	IL-6R rs12083537; rs11265618	Tocilizumab	rs12083537 A/A and rs11265618 C/C were associated with better EULAR response
Luxembourger et al. 2019	IL-6R rs12083537	Tocilizumab	rs12083537 A/A was associated with better response than A/G or G/G
Jančić et al. 2013	IL-6 −174	Etanercept	IL-6 −174 G/G was associated with better response than G/C or C/C
Davila-Fajardo et al. 2014	IL-6 −174	Infliximab–Adalimumab–Etanercept	IL-6 −174 G/G was associated with better response than G/C or C/C
Ruyssen-Witrand et al. 2012	FCGR3A −158	Rituximab	FCGR3A −158V allele was associated with a better response
Quartuccio et al. 2014	FCGR3A −158	Rituximab	The FCGR3A −158 V/V G was associated with better response than V/F or F/F
Pai et al. 2017	FCGR3A −158	Rituximab	The FCGR3A −158 V/V and V/F were associated with better response than F/F
Jiménez Morales et al. 2019	FCGR2A 9541 FCGR3A 10872	Rituximab	The FCGR2A 9541TT genotype was associated with higher EULAR response. The FCGR3A 10,872 G allele was associated with a greater improvement in DAS28 score
Ruyssen-Witrand et al. 2013	BAFF −871C	Rituximab	BAFF −871 C/C was associated with a better response than T/T
Camp et al. 2015	IL-1α (+4845) IL-1β (+3954)	Anakinra	Haplotype IL-1α (+4845) rare allele IL-1β (+3954) rare allele was associated with a higher response
